# From a variant of unknown significance to pathogenic: Reclassification of a large novel duplication in *BRCA2* by high‐throughput sequencing

**DOI:** 10.1002/mgg3.1045

**Published:** 2019-11-13

**Authors:** Jana Lisa van Luttikhuizen, Janin Bublitz, Stephanie Schubert, Gunnar Schmidt, Winfried Hofmann, Susanne Morlot, Reena Buurman, Bernd Auber, Brigitte Schlegelberger, Doris Steinemann

**Affiliations:** ^1^ Department of Human Genetics Hannover Medical School Hannover Germany

**Keywords:** BRCA2, copy number variation, hereditary breast and/or ovarian cancer, high‐throughput sequencing, large genomic rearrangement

## Abstract

**Background:**

Germline mutations in *BRCA1*/*2* significantly contribute to hereditary breast and/or ovarian cancer. Here, we report a novel *BRCA2* duplication of exons 22–24 in a female patient with bilateral breast cancer at age 35 and 44. The duplicated region was initially detected by gene panel sequencing and multiplex ligation‐dependent probe amplification. However, the location and orientation of the duplicated region was unknown. Therefore, it was initially classified as a variant of unknown significance.

**Methods:**

The spatial directional characterization of the *BRCA2* duplication was achieved by targeted enrichment of the whole‐genomic *BRCA2* locus including exons and introns, and subsequent high‐throughput sequencing. Subsequently, bioinformatics tools and a breakpoint‐spanning PCR were used for identification of location and orientation of the duplication.

**Results:**

The duplicated region was arranged in tandem and direct orientation (Chr13(GRCh37):g.32951579_32960394dup; NM_000059.3 c.8754 + 651_9256+6112dup p.(Ala3088Phefs*3)). It is predicted to result in a frameshift and a premature stop codon likely triggering nonsense‐mediated mRNA decay. Consequently, it is regarded as pathogenic.

**Conclusion:**

This case study demonstrates that a comprehensive characterization of a structural variant by breakpoint assessment is crucial for its correct classification. Therefore, sequencing strategies including non‐coding regions might be necessary to identify cancer predispositions in affected families.

## Graphical abstract

In our manuscript we report a novel *BRCA2* duplication of exons 22–24 in a female patient with bilateral breast cancer. The duplicated region was initially detected by gene panel sequencing and multiplex ligation‐dependent probe amplification. Since the breakpoints are located in introns, the duplication could only be further characterized by high‐throughput sequencing including noncoding regions.

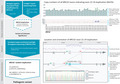

1

The well‐known tumor suppressor genes *BRCA1/2* (MIM #113705/#600185) are crucial to restore genomic stability after DNA double‐strand breaks via homologous recombination. Germline pathogenic variants (PVs) in one of the genes significantly increase the lifetime risk to develop hereditary breast and/or ovarian cancer (HBOC, MIM #604370). About 25% of HBOC can be explained by pathogenic *BRCA1/2* variants (Nielsen, van Overeem Hansen, & Sorensen, 2016). Most *BRCA1/2* PVs described in literature are single‐nucleotide variants. However, 12.42% of *BRCA1* PVs and 4.17% of *BRCA2* PVs are large genomic rearrangements (LGRs) (Lopez‐Urrutia et al., 2018). The increased occurrences of *BRCA1* LGRs compared to *BRCA2* is most likely due to its higher density of *Alu* repeat sequences, which are susceptible to homologous recombination (Woodward, Davis, Silva, Kirk, & Leary, 2005). As a consequence, the detection of LGRs performed in diagnostics is initially recommended to include only *BRCA1* and has been extended to *BRCA2* in recent years (Engert et al., 2008).

A widely used method to detect LGRs is multiplex ligation‐dependent probe amplification (MLPA), which has been first introduced in 2003. MLPA is a semi‐quantitative PCR‐based technique that can determine the copy numbers of different target sequences, simultaneously (Hogervorst et al., 2003). Although the technique has been proven to be effective and sensitive in the detection of copy number variations (Belvini, Salviato, Radossi, & Tagariello, 2017; Scaglione et al., 2018), it cannot identify the position or orientation of duplications within the genome (Rost, Loffler, Pavlova, Muller, & Oldenburg, 2008). Therefore, conclusive characterization regarding the pathogenicity of duplications is impossible using solely MLPA. In these cases, additional methods are required for the classification of variants. Before high‐throughput sequencing was accessible, MLPA has often been combined with a breakpoint‐spanning PCR and a Sanger sequencing‐based primer walk for the identification of the exact breakpoint. Although the method is simple and inexpensive, it can be labor‐intensive, when the region that needs to be covered is large and the orientation is unknown.

Nowadays, high‐throughput sequencing is a feasible option to detect genomic rearrangements (Nunziato et al., 2017). However, panel or whole‐exome sequencing does not cover intronic or intergenic regions of the genome, while whole‐genome sequencing is often not the most cost‐effective method. Here, we used a targeted enrichment strategy, which allowed the high‐throughput sequencing of selected regions, including exons and introns. We were able to identify a novel tandem duplication of exons 22–24 of *BRCA2*, which is most likely causative for HBOC.

The patient (III:7) (Figure 1) was first diagnosed with an invasive ductal carcinoma of the right mammary gland at the age of 35 (pT1c (2), N1, G3, estrogen receptor (ER) positive, progesterone receptor (PR) negative, and human epidermal growth factor receptor 2 (HER2) negative). After performing right mastectomy, the patient received adjuvant chemotherapy (4 × epirubicin/cyclophosphamide, 4 × Taxol), and tamoxifen for 3 years. With 44 years of age, she was diagnosed with a moderately differentiated invasive mammary carcinoma in her left mammary gland (pTX, G2, ER positive, PR positive, and HER2 negative).

**Figure 1:**
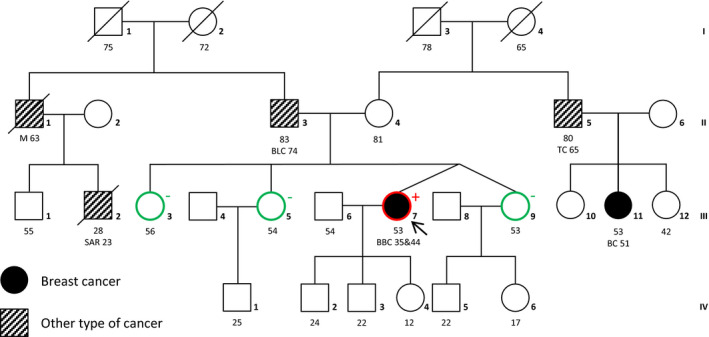
Family pedigree. Circles: female; squares: male; filled solid: breast cancer; striped: other type of cancer; unfilled: unaffected; red outline: *BRCA2* duplication carrier; green outline: non‐carrier; black outline: not examined. BC, breast cancer; BBC, bilateral breast cancer; BLC, bladder cancer; TC, testicular cancer; M, Melanoma; SAR, sarcoma. The numbers below the symbols represent the age of the individual, followed by type of cancer and age at cancer onset. The index patient is marked by an arrow

Within the patient's family, multiple incidences of different cancer types appeared, including one other incidence of breast cancer (Figure 1). Her maternal first cousin (III:11) developed a tubular and papillary mammary gland carcinoma at the age of 51. Her maternal uncle (II:5) was diagnosed with testicular cancer at the age of 65. The patient's father (II:3) was diagnosed with bladder cancer at the age of 74. His brother had an unidentified primary tumor, most likely a melanoma, which metastasized into the brain and the neck, where it formed a clearly visible lymph node tumor. He died at the age of 63. His son (III:2) died at the age of 28 as a result of a fibrosarcoma in his thigh diagnosed 3 years earlier.

The proband's young age at the initial breast cancer diagnosis and the development of bilateral breast cancer before the age of 50, suggested a genetic predisposition for the disease. In 2010, after the second incidence of breast cancer, she was first examined in our clinic. Genomic DNA was isolated from EDTA blood samples using the QIAamp DNA blood Midi kit (Qiagen) and was tested for single‐nucleotide variants in *BRCA1/2* via high‐resolution melt (HRM) analysis and denaturing high‐performance liquid chromatography (DHPLC). Additionally, she was tested for CNVs in *BRCA1* via MLPA (SALSA® MLPA® P002 *BRCA1* Kit, MRC‐Holland). No PV was identified.

Since other types of cancer occurred in the family, including sarcoma, the patient was tested for Li‐Fraumeni syndrome (MIM #151623). In approximately 70% of families fulfilling the criteria for classical Li‐Fraumeni syndrome, a PV in *TP53* can be identified (Evans et al., 2002). In 2018, screening of *TP53* was performed via the TruSight Cancer Panel (Illumina). On this occasion, MLPA testing for *BRCA2* (SALSA® MLPA® P045 *BRCA2*/*CHEK2* Kit, MRC‐Holland) was also performed, which was implemented as a routine screening in our clinic in 2012. Both techniques detected a duplication of exons 22–24 of *BRCA2*, but its location and orientation within the genome of the duplicated region remained unknown. Due to the large introns surrounding exon 22 and exon 24, determining the duplication's orientation and breakpoints using PCR‐based sequencing techniques would have been labor‐intensive and inefficient. Therefore, the duplication has initially been classified as a variant of unknown significance (VUS), according to the ENIGMA classification criteria for *BRCA1/2* variants (Version 2.5.1 29 June 2017).

To further characterize the duplication, the complete *BRCA2* locus of the patient was amplified via a custom‐designed targeted enrichment strategy (Agilent SureSelect^QXT^) and sequenced on a NextSeq 500 (Illumina) according to the manufacturer's instructions. Briefly, 50 ng of the patient's genomic DNA was enzymatically fragmented and adaptor‐tagged. The DNA fragments of interest hybridized to biotin‐labeled cRNA baits targeting the genomic loci of *BRCA1* (Chr17(GRCh37):g.40600254_41756008) and *BRCA2* (Chr13(GRCh37):g.32414335_33482189), including exonic, intronic, and surrounding intergenic regions. The cRNA‐DNA hybrids were captured using streptavidin‐coated magnetic beads. After washing and amplification, the enriched DNA fragments were submitted to paired‐end sequencing using a NextSeq 500 Mid Output V2 kit for 2 × 150 bp paired‐end reads (Illumina). A mean coverage of 422 reads with 99.61% of bases covered by at least 20 reads was achieved.

The in silico copy number variation detection tool CnvHunter (https://github.com/imgag/ngs-bits) confirmed the duplication covering exons 22–24 of *BRCA2* (Figure 2). The tool detects duplications and deletions by comparing the coverage profiles of samples (*n* = 80) sequenced with the same targeted enrichment method. By analyzing the reads, we were able to determine the breakpoint and its direct tandem orientation. Additionally, a breakpoint‐spanning PCR was performed, using the following primers for validation, 5′‐CTCTAGAGTACTGTGAGTGG‐3′ (Chr13(GRCh37):g.32960177–32960196) and 5′‐GAGACGGAGTTTTGCTCTTG‐3′ (Chr13(GRCh37):g.32951708–32951727). The breakpoints of the duplication were located within the homologous region TAAAAATACAAAA: Chr13(GRCh37):g.32951579_32960394dup (Figure 2). This tandem duplication of exon 22–24 (about 8,815 bp; HGVS: NM_000059.3 c.8754 + 651_9256+6112dup p.(Ala3088Phefs*3)) is predicted to cause a frameshift and a premature termination codon, which is located more than 50 nucleotides upstream of the last exon–exon junction. This is known to be critical to trigger nonsense‐mediated mRNA decay (Lindeboom, Supek, & Lehner, 2016). Therefore, we regard this variant to be pathogenic.

**Figure 2:**
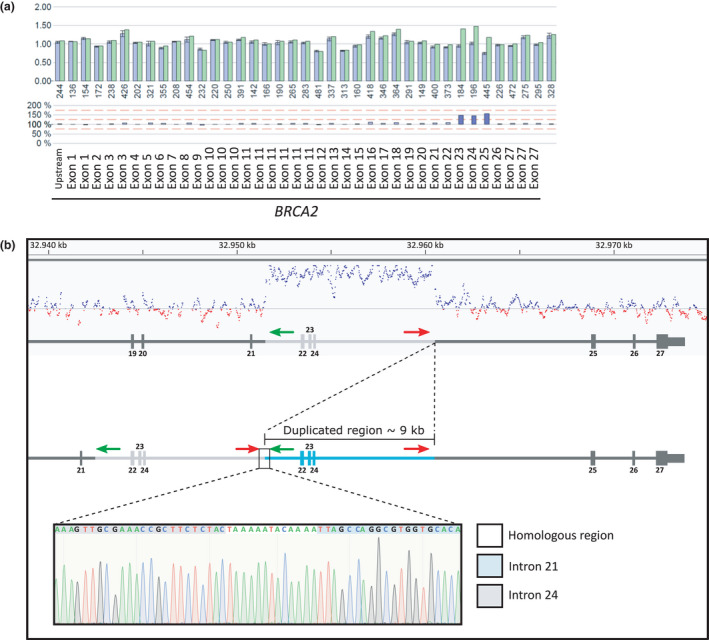
Schematic overview of the duplicated region of *BRCA2* exons 22–24. (a) MLPA analysis showing *BRCA2* dosage of the proband. Upper histogram: mean relative peak area (RPA) of three references (blue bars) and the proband (green bars) with standard deviations. The amplicon size (bp) of each probe is indicated below. Bottom histogram: differences of RPA between the proband and the references in percentage. The mean RPA of the references was normalized to 100%. Differences higher than 125% are demonstrated by dark blue bars. The location of each probe is marked below. (b) The duplication and its genomic location detected by CnvHunter are depicted in the upper panel. Below the CnvHunter results, the normal exon‐intron structure of *BRCA2* (NM_000059.3) and the exon‐intron structure including the tandem duplication (blue) are shown. The Sanger sequencing chromatogram of the breakpoint amplified by the breakpoint‐spanning PCR demonstrates the last nucleotides of intron 24 (gray), a homologous region, where the genomic break occurred (white) and the first nucleotides of intron 21 (blue). Binding sites of forward (5′‐CTCTAGAGTACTGTGAGTGG‐3′ (Chr13(GRCh37):g.32960177–32960196)) and reverse (5′‐GAGACGGAGTTTTGCTCTTG‐3′ (Chr13(GRCh37):g.32951708–32951727)) primer as used for the breakpoint‐spanning PCR are represented by red and green arrows, respectively

Considering this classification, the patient's sisters (Figure 2; III:3, 5 and 9) were tested for the genomic duplication via Sanger sequencing. All of them were negative for the *BRCA2* duplication. Unfortunately, additional family members including the proband's parents did not consent to genetic testing for the duplication. Therefore, it is unknown whether this duplication emerged de novo in the patient. The patient was advised to undergo a risk‐reducing salpingo‐oophorectomy. An earlier discovery of the *BRCA2* duplication might have ultimately resulted in the same therapy, but a bilateral mastectomy would have been offered.

Here, we demonstrate the rapid and accurate identification of a large genomic rearrangement via a targeted high‐throughput sequencing approach. With this method, we were able to reclassify a variant of unknown significance to a pathogenic variant. This highlights the importance of thorough characterization of LGRs, such as duplications, by the identification of their location and orientation to determine their pathogenicity. This might also require targeting intronic regions, which are mostly excluded in high‐throughput sequencing panels. In this context, targeted enrichment is a powerful and cost‐effective tool for complete coverage of target regions and the characterization of large genomic rearrangements.

## CONFLICT OF INTEREST

The authors declare that they have no competing interests.

## AUTHOR CONTRIBUTIONS

J.L.v.L., J.B., and S.S. performed the high‐throughput sequencing, analyzed the data, wrote, and revised the manuscript; G.S. analyzed the data and provided scientific advice; W.H. performed the bioinformatics analysis; S.M. contacted the patients; R.B. analyzed the MLPA results; B.A., B.S., and D.S. revised the manuscript; D.S. supervised the work.

## Data Availability

The datasets generated and/or analyzed during the current study are not publicly available but are available from the corresponding author on reasonable request.
